# Properties of bootstrap tests for *N*‐of‐1 studies

**DOI:** 10.1111/bmsp.12071

**Published:** 2016-10-06

**Authors:** Sharon X. Lin, Leanne Morrison, Peter W. F. Smith, Charlie Hargood, Mark Weal, Lucy Yardley

**Affiliations:** ^1^ Southampton Statistical Sciences Research Institute (S3RI)University of SouthamptonUK; ^2^ National Institute for Health Research (NIHR) Wessex Collaboration for Leadership and Research in Health Care (CLAHRC)University of SouthamptonUK; ^3^ Academic Unit of PsychologyUniversity of SouthamptonUK; ^4^ Electronics and Computer ScienceUniversity of SouthamptonUK

**Keywords:** *N*‐of‐1 studies, power, semi‐ and parametric bootstrapping, Wald test, Type I error rate

## Abstract

*N*‐of‐1 study designs involve the collection and analysis of repeated measures data from an individual not using an intervention and using an intervention. This study explores the use of semi‐parametric and parametric bootstrap tests in the analysis of *N*‐of‐1 studies under a single time series framework in the presence of autocorrelation. When the Type I error rates of bootstrap tests are compared to Wald tests, our results show that the bootstrap tests have more desirable properties. We compare the results for normally distributed errors with those for contaminated normally distributed errors and find that, except when there is relatively large autocorrelation, there is little difference between the power of the parametric and semi‐parametric bootstrap tests. We also experiment with two intervention designs: ABAB and AB, and show the ABAB design has more power. The results provide guidelines for designing *N*‐of‐1 studies, in the sense of how many observations and how many intervention changes are needed to achieve a certain level of power and which test should be performed.

## 1. Introduction

*N*‐of‐1 study designs involve the collection and analysis of repeated measures of an individual unit using an intervention and not using an intervention. The design for an *N*‐of‐1 study is often called the single case experiment design or single subject experiment design. The data from *N*‐of‐1 studies typically consist of *T* repeated measures, *y*_*t*_, *t* = 1, …, *T*, from a single subject, and dummy variables, *x*_*t*_, indicating whether or not there is an intervention at time *t*. The ultimate goal of *N*‐of‐1 studies is to investigate the effect of an intervention on an individual unit, and they have been applied in areas such as psychology and education (Shadish & Sullivan, 2011), and medicine (Howick *et al*., 2011).

Over the years, various analysis methods for *N*‐of‐1 studies have been developed and modified for more effective and simpler approaches to detecting intervention effects between periods that are subject to no interventions (phase A) and those that are subject to interventions (phase B). By and large these methods can be divided into two categories: non‐regression‐based (Borckardt, Nash, Murphy, Moore, Shaw, & O'Neil, 2008; Nourbakhsh & Ottenbacher, 1994; Parker, Vannest, & Brown, 2009); and regression‐based (Huitema & McKean, 2000; McKnight, McKean, & Huitema, 2000). The former methods are simpler and easier to implement without formal statistical modelling, while the latter are based on regression theory, where parameters are formally estimated. Given the increasing adoption of *N*‐of‐1 studies for evidence‐based analyses (Kratochwill *et al*., 2013), we concentrate on regression‐based methods in this study. In particular, we estimate the statistical power of semi‐parametric and parametric bootstrap tests under two single case designs, aiming to address the issue of lack of power analyses in the current literature.

We use a sample collected by a mobile phone app called “POWeR Tracker” (Morrison *et al*., 2014) to illustrate the power of the Wald test and bootstrap tests. Table 1 lists an extract of the data from an *N*‐of‐1 study to understand the impact on physical activity levels of using a smartphone application for weight management. It is a record of total steps of one participant over the period of 28 days. It has an ABAB experimental design (7 days without, 7 days with, 7 days without, 7 days with an intervention). In phase A, the participant had access to a web‐based intervention (POWeR) only. In the intervention phase (phase B), the participant had access to both the web‐based intervention and app‐based intervention (POWeR tracker). During both phases, daily steps were recorded via a blinded pedometer.

**Table 1:** An extract of total daily steps of one individual user not using (phase A) and using (phase B) the POWeR Tracker app

Day	Total steps	POWeR Tracker phases
1	NA	A
2	11,471	A
3	9,760	A
4	3,558	A
5	4,739	A
6	3,662	A
7	NA	A
8	5,729	B
9	2,794	B
10	7,636	B
11	3,996	B
12	7,467	B
13	10,587	B
14	3,863	B
15	1,649	A
⋮	⋮	⋮
20	3,566	A
21	3,457	B
⋮	⋮	⋮
28	6,335	B

Note

NA, missing data.

**Figure 1:**
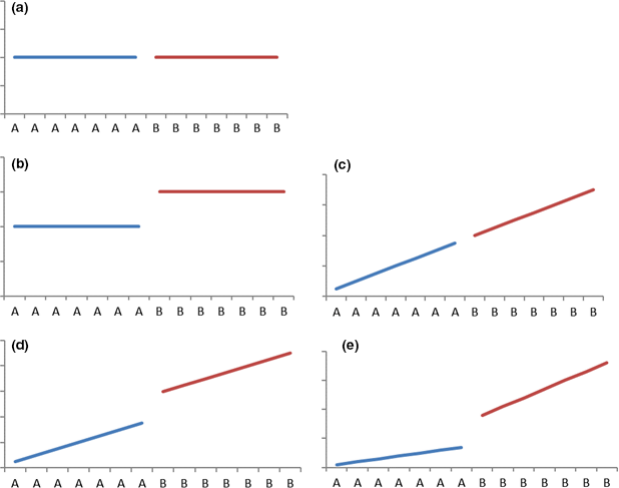
Patterns of mean behaviour. ‘A’ (blue line) and ‘B’ (red line) refer to a phase without an intervention and with an intervention, respectively. [Colour figure can be viewed at www.online library.com].

We initially consider the following general regression model for an *N*‐of‐1 study:

(1) $$y_{t}=\beta_{0}+\beta_{1}x_{t}+\beta_{2}t+\beta_{3}tx_{t}+\epsilon_{t},$$

where $\epsilon_{t}=\rho \epsilon_{t-1}+z_{t},t=2,\ldots ,T,$ are autocorrelated, order‐one residuals, with *z*_*t*_ ~ *N*(0, σ^2^), and $\epsilon_{1}$ ~ *N*(0, σ^2^/(1 − ρ^2^)). Before formally discussing the methodology, we introduce five possible alternative underlying mean behaviour patterns for two phases which can be specified by model (1) (Figure 1). A is the phase before an intervention and B is the phase after an intervention has been introduced. In Figure 1a, there is no change in the intercept or slope following the intervention (β_1_ = β_2_ = β_3_ = 0). In Figure 1b and d there is a change in the intercept but not in the slope (β_1_ ≠ 0, β_3_ = 0). The difference between these two figures is that the former has a zero slope (β_2_ = 0). In Figure 1c, there is constant increase over time, that is, no change in the slope (β_1_ = 0, β_2_ ≠ 0, β_3_ = 0). No intervention changes could be detected in this figure since it is a trend developed in phase A continuing into phase B. Figure 1e represents a change in both the intercept and slope (β_1_ ≠ 0, β_2_ ≠ 0, β_3_ ≠ 0).

A regression‐based *N*‐of‐1 study analyses a single interrupted time series that is subject to no interventions and interventions. It has two common methodological difficulties: autocorrelation and a small sample. McKnight *et al*. (2000) designed a double bootstrap methodology to tackle autocorrelation bias in the context of small samples. They use the first bootstrap to obtain asymptotically consistent estimates of the autocorrelation and other parameters in the model by utilizing Durbin's two‐stage method, and use the second bootstrap to estimate the variance–covariance matrix of the estimated parameters. Their method reduced biases in the estimated autocorrelation and standard errors of the coefficients, and hence provided tests that have Type I error rates closer to the nominal rate and comparable statistical power to that when the true value of the autocorrelation is used. However, their estimation process is extremely computer‐intensive by construction, which may limit the potential applications of method in practice. The current study attempts to deal with the issues of autocorrelation and small sample using a single parametric bootstrap within a generalized least squares (GLS) framework. Our work uses the restricted maximum likelihood (REML) estimation method in R (R Development Core Team, 2014) to detect an effect between two phases (phase A has no intervention, phase B has an intervention) where the underlying data series is autocorrelated. Parameters estimated under a GLS approach are consistent, but may suffer bias from underestimated standard errors (Park & Mitchell, 1980) due to the small sample size. We use semi‐parametric and parametric bootstrap tests to reduce the effect of small sample bias in test statistics in an attempt to achieve better inferences from estimated parameters than the Wald test. Our method provides a simpler option that deals with the autocorrelation and small sample issues. It is less computer‐intensive and easier to implement when compared to the double bootstrap method.

Motivated by Borckardt *et al*. (2008), we consider a simple case design that explicitly assumes there is no slope in our model and hence concentrates on the differences among two phases (A and B). This is a realistic assumption as empirical experiments may not have a trend in phase A or B (see our motivating example). Our null hypothesis is displayed in Figure 1 and does not include a trend. Our alternative hypothesis is in Figure 1b. We use a dummy variable to detect a phase effect between A and B in one single time series as in standard linear regression analysis. A dummy variable that is not significantly different from zero indicates there is no phase effect. Further, we use simulation to calculate and compare statistical properties of bootstrap tests and Wald tests under various autocorrelations and phase effects. Despite new methods continually being developed to carry out *N*‐of‐1 studies, there is limited evidence on the power of these tests. This is the first attempt, to the best of our knowledge, to investigate the statistical power of semi‐parametric and parametric bootstrap tests within a single time series setting in the context of *N*‐of‐1 studies. The results on statistical power provide guidelines for designing *N*‐of‐1 studies, in the sense of how many days and how many intervention changes are needed to achieve a certain level of power.

The rest of the paper is organized as follows. Section 2. introduces the regression model for detecting phase effects, the concepts of Type I error rate and statistical power, the construction of bootstrap tests and the estimation of the Type I error rate and power functions. Section 3. presents empirical results from two intervention designs (AB and ABAB), a discussion of these results and a power function illustration using the sample data introduced above. Our conclusions are summarized in Section 4.1.

## 2. Methodology

### 2.1 Regression model

We now consider a simpler version of model (1) for an *N*‐of‐1 study:

(2) $$y_{t}=\alpha +\beta x_{t}+\epsilon_{t},$$

where $\epsilon_{t}=\rho \epsilon_{t-1}+z_{t},t=2,\ldots ,T,$ with *z*_*t*_ ~ *N*(0, σ^2^), and $\epsilon_{1}$ ~ *N*(0, σ^2^/(1 − ρ^2^)). Recall that in this model, *y*_*t*_ is a repeated measure at time *t *=* *1, 2, …, *T*,* x*_*t*_ is a phase dummy taking the value of 1 for the intervention and 0 for the non‐intervention phase. The phase effect is β, with a large (small) absolute value of β indicating a large (small) phase effect.

As mentioned, the problems of small sample size and autocorrelation may violate the underlying assumptions of no autocorrelation and large sample size for a standard linear regression analysis, which may lead to incorrect inferences, such as an incorrect Type I error rate and low statistical power. In order to overcome the problem of autocorrelation, we 'use GLS with REML to fit the models. Motivated by McKnight *et al*.'s (2000) bootstrap method, in order to address the small‐sample problem, we suggest constructing semi‐parametric and parametric bootstrap tests of the null hypothesis H_0_: β = 0. For these tests, rather than comparing the Wald test statistics to its asymptotic null distribution, *N*(0,1), which henceforth we refer to as the Wald test, we compare this test statistics to a bootstrapped sample. See Section 2.3 for details. We compare the properties of the bootstrap tests to those of a Wald test for coefficients estimated by using GLS with REML. The properties under investigation are the Type I error rate and statistical power. By doing so, we aim to uncover the actual magnitude of Type I error rate, and how close it is to the nominal rate of 5%. Both Wald tests and bootstrap tests are carried out using the data simulated as described in Section 3.. All estimates are calculated using the GLS REML routine in R (R Development Core Team, 2014).

### 2.2 Statistical properties

Two properties of a statistical test are discussed in this study: the Type I error rate and statistical power. The Type I error rate is the probability of incorrectly rejecting the null hypothesis when it is true. It is an important property which we like to control accurately. A standard acceptable Type I error is 5%.

Statistical power, or the power of a significance test, refers to the probability of rejecting the null hypothesis when it is false. Given a valid procedure, we like the power as high as possible when the null hypothesis is false (Cohen, 1988). It is an important consideration in an *N*‐of‐1 study and gives guidance on the length and frequency of interventions to reach a desirable power level, such as 80% (Cohen, 1988). It can also be used to detect whether two or more individuals are required in the trial (*N*‐of‐*k* studies).

### 2.3 Construction of bootstrap tests

The concept of the bootstrap is to replace the population with the empirical population (non‐parametric) or estimated population (semi‐parametric and parametric). Suppose our target is to draw inference about a population parameter θ and we have observed a random sample of size *T* (*y*_1_, *y*_2_, … *y*_*T*_) from this population with sample statistics $\hat{\theta }$. We can derive ${\hat{\theta }}_{b}^{*}$, a random quantity which represents the same statistics, but computed on a bootstrap sample *b* drawn from the empirical or estimated population. Computing ${\hat{\theta }}_{b}^{*}$ for *B* different bootstrap samples, we can then derive ${\hat{\theta }}_{1}^{*}$, ${\hat{\theta }}_{2}^{*}$, … ${\hat{\theta }}_{B}^{*}$. The empirical bootstrap distribution of ${\hat{\theta }}_{b}^{*}$ proves to be a fairly good approximation of the distribution of $\hat{\theta }$.

In this study, we adopt parametric bootstrap tests (Efron & Tibshirani, 1993, p. 53) and semi‐parametric bootstrap tests (Davison & Hinkley, 1997, pp. 389–391), with *B *=* *100. Since we are not estimating a *p*‐value, just performing a bootstrap test, *B* = 100 should be sufficient. However, to assess the effect of using a large *B*, we also calculate the Type I errors for bootstrap tests with *B* = 200 and compare the results. Under the alternative hypothesis as in model (2), the ${\hat{\alpha }}_{A}$, ${\hat{\beta }}_{A}$, ${\hat{\rho }}_{A}$ and ${\hat{\sigma }}_{A}^{2}$ from the observed sample are estimates of population values of α, β, ρ and σ^2^. We also estimate ${\hat{\alpha }}_{0}$, ${\hat{\rho }}_{0}$ and ${\hat{\sigma }}_{0}^{2}$ from the observed sample under the null hypothesis that β is zero, that is, under the following model:

(3) $$y_{t}=\alpha +\epsilon_{t},$$

where $\epsilon_{t}=\rho \epsilon_{t-1}+z_{t},t=2,\ldots ,T,$ with *z*_*t*_ ~ *N*(0, σ^2^), and $\epsilon_{1}$ ~ *N*(0, σ^2^/(1 − ρ^2^)).

For the parametric tests, we simulate bootstrap samples under the null hypothesis. Then model (2) is fitted to the bootstrap samples to generate bootstrap estimates of β (${\hat{\beta }}_{1}^{*}$, ${\hat{\beta }}_{2}^{*}$, …, ${\hat{\beta }}_{B}^{*}$). For each of the bootstrap simulations, the absolute value of the Wald test statistic based on ${\hat{\beta }}_{b}^{*}$ is compared to the Wald test statistics based on $\hat{\beta_{A}}$ estimated under the alternative hypothesis (model (2)). This comparison is repeated *B* times for each of the simulated ${\hat{\beta }}_{b}^{*}$. The *p*‐value of the bootstrap test is calculated as the percentage of times out of the total *B* that the bootstrapped Wald statistics generated from model (3) is more extreme than the observed statistics from model (2).

For the semi‐parametric test, rather than simulating the errors, *z*_*t*_, from a normal distribution, they are sampled with replacement from the estimate residuals from model (3): ${\hat{z}}_{t}={\hat{\epsilon }}_{t}-\hat{\rho }{\hat{\epsilon }}_{t-1}$, *t* = 2, …, *T*, transformed to have mean zero and variances ${\hat{\sigma }}_{0}^{2}$; see Davison and Hinkley (1997, pp. 389–391) for more details.

### 2.4 Estimating the Type I error rate and power functions

We estimate and compare the properties of bootstrap tests and those of the Wald tests. The Type I error rate and the power function of both tests are estimated. As mentioned, we desire the actual Type I error rate to be close to nominal rate of 5% and high statistical power. We start by simulating a data set *Y*_*t*_, *t *=* *1, …, *T*, following model (2), with predetermined values of α, β, ρ and σ^2^, where α = 0, σ^2^ = 1, and follow the structure of data collected from a study of POWeR Tracker (Morrison *et al*., 2014), with an ABAB design. Wald and bootstrap tests are then performed on the simulated data. We repeat this process 10,000 times for the Wald test and the bootstrap tests, and estimate the Type I error rate and power function for the given β and ρ. The actual Type I error rate is estimated as the percentage of times that the *p*‐values of estimated $\hat{\beta }$ are <5% when β is set to zero. The statistical power is the percentage of times that the *p*‐values are <5% when β is not zero. A power function is the power as the corresponding values of β and ρ vary.

We expect the Type I error to be close to the nominal size of 5% for the parametric bootstrap test we have constructed. We also expect low statistical power in our study as autocorrelation and the small sample in *N*‐of‐1 studies are long‐standing issues in behaviour change research in psychology (Cohen, 1988).

In order to assess the impact of the normal assumption, we also simulate the residuals from a contaminated normal distribution with a random 15% of the residual generated with an increased variance of 25 and repeat the simulation study. However, the parametric bootstrap was still based on the assumption of normality as before.

## 3. Simulation study

The Type I error rate and statistical power functions for both the Wald test and the parametric bootstrap tests are estimated by using Monte Carlo simulation. As noted above, we use 10,000 simulations for all tests and *B *=* *100 for the bootstrap tests. Two designs of interventions are calculated: the first design (D1), as in the POWeR Tracker study, has an ABAB structure, with each of the four phases set up for 7 days (as in column 3 in Table 1); the second design (D2) has an AB structure, with both phases lasting for a period of 14 days. Both designs have a total duration of 28 days. The results give guidance on the design of *N*‐of‐1 studies, in the sense of whether it is better to have one long intervention period or several shorter intervention periods.

### 3.1 Type I error rates

Table 2 presents the estimated Type I error rates for the Wald, parametric and semi‐parametric tests. Four values of ρ are considered (0, .2, .5, .7) with two error distributions (normal and contaminated normal) and two designs (D1 and D2). For the bootstrap tests we present the results for *B* = 100 and 200. When interpreting these estimates, it should be borne in mind that if the true proportion is .05 then under repeated sampling approximately 95% of the estimated proportions based on a sample size of 10,000 would be in the tolerance interval (.0457, .0543).

**Table 2:** Estimated Type I error rates for Wald and bootstrap tests for two intervention designs

Test	Design 1 (D1)				Design 2 (D2)			
	ρ = 0	ρ = .2	ρ = .5	ρ = .7	ρ = 0	ρ = .2	ρ = .5	ρ = .7
Normal errors								
Wald	.0674	.0728	.0854	.0808	.0612	.0697	.1000	.1130
Parametric bootstrap								
*B *=* *100	.0503	.0503	.0522	.0500	.0513	.0510	.0529	.0540
*B *=* *200	.0513	.0522	.0507	.0544	.0460	.0489	.0508	.0537
Semi‐parametric bootstrap								
*B *=* *100	.0513	.0519	.0522	.0541	.0457	.0462	.0465	.0510
*B *=* *200	.0494	.0479	.0505	.0499	.0508	.0475	.0458	.0561
Contaminated normal errors								
Wald	.0522	.0625	.0820	.0917	.0547	.0688	.1071	.1223
Parametric bootstrap								
*B *=* *100	.0434	.0483	.0475	.0559	.0411	.0448	.0615	.0698
*B *=* *200	.0382	.0411	.0494	.0612	.0391	.0425	.0552	.0677
Semi‐parametric bootstrap								
*B *=* *100	.0442	.0436	.0506	.0508	.0392	.0446	.0544	.0577
*B *=* *200	.0428	.0432	.0464	.0518	.0408	.0456	.0587	.0584

For all but one of the scenarios, the estimated Type I error rate for the Wald test is greater than the upper limit of the 95% tolerance interval (.0543), indicating that Wald test does not have the correct Type I error rates. However, for normal errors, the estimated Type I error rates for the parametric and semi‐parametric bootstrap tests are within the 95% tolerance interval.

For the contaminated errors, the majority of the estimated Type I errors for the parametric and semi‐parametric tests are closer to the nominal value of .05 than those for the Wald test, although fewer are within the 95% tolerance interval than for the normal errors. Furthermore, the estimate Type I error rates for the semi‐parametric tests are closer to the normal value than those for the parametric tests, particularly for D2 and ρ = .5 and .7, indicating that in the presence of contaminated errors, the semi‐parametric bootstrap tests perform better.

For both bootstrap tests and both error distributions, the results for *B* = 100 and 200 are very similar, supporting our initial belief that *B* = 100 should be sufficient. In particular, note that for the parametric bootstrap with contaminated errors, the Type I error rates with the larger *B* are not uniformly closer to the nominal rate than those with *B* = 100 (Table 2).

### 3.2 Power

**Figure 2:**
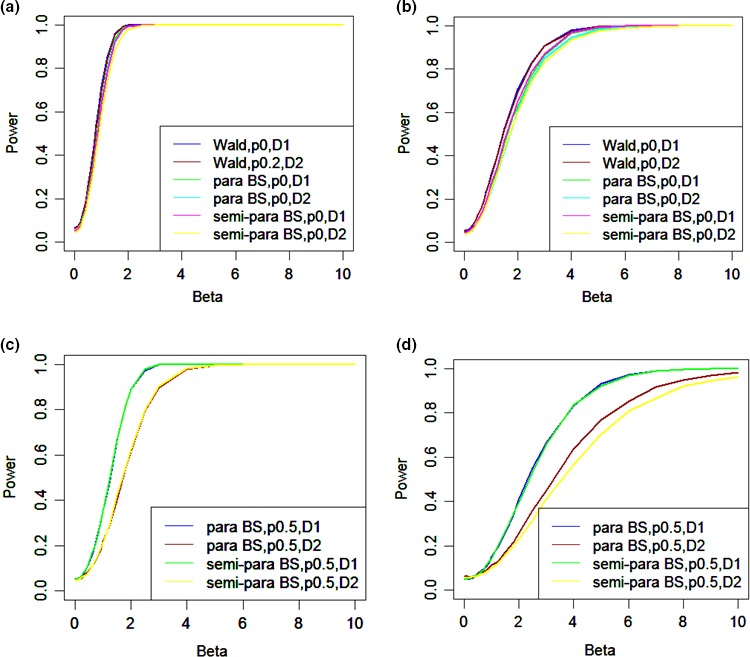
Power functions for: the Wald test, parametric test and semi‐parametric bootstrap tests under the two designs with ρ = 0 and (a) normal errors and (b) contaminated errors; the bootstrap tests under the two designs with ρ = .5 and (c) normal errors and (d) contaminated errors.

Tables A1, A2, A3, A4, A5, A6 (Appendix) present estimates of power functions for the Wald, parametric and semi‐parametric tests with *B* = 100. Four values of ρ are considered (0, .2, .5, .7) with two error distributions (normal and contaminated normal) and two designs (D1 and D2). Note that D1 has three change points, whereas D2 has only one. Figure 2 presents a range of these power functions.

Figure 2 presents the power functions of the three tests under the two designs with ρ = 0 and normal errors (Figure 2a) and contaminated errors (Figure 2b). Although the Wald has the incorrect Type I error rate, it is the scenario which is closest to the nominal rate and therefore is included for comparison. From these two graphs, we conclude that there is no substantial differences in power when ρ = 0.

Figure 2c presents the power functions of the bootstrap tests under the two designs with ρ = .5 and normal errors. Again there is no difference between the two tests for each design, but they are considerably more powerful under D1.

Figure 2d presents the same power functions as in Figure 2c, except that the errors are now contaminated. Again there is no difference between the parametric and semi‐parametric bootstrap test under D1, whereas under D2 the semi‐parametric test is less powerful. However, recall that in this case the semi‐parametric test has estimated Type I error rates closer to the nominal.

Comparing Figure 2a and b with Figure 2c and d reveals that the power decreases as ρ increases. Inspection of Tables A1, A2, A3, A4, A5, A6 reveals this is the case in all the scenarios considered. Comparing the two designs reveals that the power is lower for the contaminated errors; again see also Tables A1, A2, A3, A4, A5, A6.

For D2 with an autocorrelation value of .2 and normal errors, to achieve a power of .8 for the parametric bootstrap test a β‐value >1.5 is required; for a larger autocorrelation value, .5 or .7, a β of 2.5 or 3 is required (Table A3). These results suggest the power under D2 is low. Further comparison between D1 and D2 reveals that the bootstrap tests for both designs generally have similar Type I error rate, but for D1 they are at least as powerful as for D2 and tend to become more powerful as ρ or β increases. This result indicates that the shorter repeated intervention design works better than the longer period of intervention without repeat. This may be due to the impact of autocorrelation.

### 3.3 Bias in $\hat{\rho }$

As a by‐product of the simulation study, we are able to assess the bias in $\hat{\rho }$ as ρ, β, the design and the error distribution vary. As expected, inspection of the results showed that the bias did not vary with β. Therefore, in Table 3, we present the estimated bias in $\hat{\rho }$ as ρ, the design and the error distribution vary. For a particular value of ρ, the bias in $\hat{\rho }$ is very similar for both designs and both error distributions. However, the magnitude of the bias increases as ρ increases. This may in part explain the inflated Type I error rates for the Wald test (Table 2). However, as noted above, the bootstrap tests perform well, despite this increase in bias.

**Table 3:** Estimated bias in $\hat{\rho }$ for two intervention designs

Errors	Design 1 (D1)				Design 2 (D2)			
	ρ = 0	ρ = .2	ρ = .5	ρ = .7	ρ = 0	ρ = .2	ρ = .5	ρ = .7
Normal	.0006	−.0146	−.0449	−.1234	.0017	−.0122	−.0396	−.1218
Contaminated	.0035	−.0060	−.0325	−.1120	.0054	.0000	−.0231	−.1085

## 4. Discussion

It is clear from the above study that the bootstrap tests are more desirable for *N*‐of‐1 studies when autocorrelation is present. Under a single case design involving one individual over a period of 28 days, the statistical power is low. The comparison between ABAB and AB designs indicates that under the presence of autocorrelation, shorter and repeated interventions (ABAB design) seem to be more effective than longer and unrepeated interventions (AB design). This result lends support for the single‐case intervention research design standards (Kratochwill *et al*., 2013) where the AB design does not meet the standard.

### 4.1 Conclusions

This study explores the properties of semi‐parametric and parametric bootstrap tests in a single subject experiment design, or *N*‐of‐1 study, aiming to account for small sample sizes under the GLS regression framework. This is the first attempt, to the best of our knowledge, to examine the properties of *N*‐of‐1 studies in such a setting. We find the bootstrap tests are more accurate with regard to Type I errors when compared to the Wald test, and hence more desirable. We recommend the use of a parametric bootstrap with *B* = 100, except when both relatively large autocorrelation and contaminated normally distributed errors are thought possible. Our results can also be used to facilitate various experimental designs and provide guidelines for future *N*‐of‐1 studies. Further, we compare two different intervention designs of the same total duration and find that the tests under the design with more change points (D1) have better properties. This provides support for designs with three changes in the intervention as set out in the single case intervention research design standards (Kratochwill *et al*., 2013).

The bootstrap methods used in the study examine an intervention effect under the assumption of no trend (Figure 1a). They can also be applied to the case where the model under the null hypothesis includes a trend (Figure 1c) and the model under the alternative hypothesis has a trend and a phase effect on the intercept (Figure 1d). Therefore, the results in this paper can also be used when designing a study to detect a phase effect on the intercept irrespective of whether or not there is a trend. The scenario our study has not covered is the case where the model under the alternative hypothesis has a phase effect on the slope (Figure 1e), although the method could easily be modified to handle this situation by adding a trend and trend by phase interaction to model (1). The method could also be extended to the situation where more than one individual is studied (*N*‐of‐*k* study, *k* > 1) by appropriately modifying model (1) to account for between‐individual differences.

## Appendix 1 Statistical power tables

### 1.1

**Table A1:** Statistical power for Wald tests under normally distributed residuals for two intervention designs

β	Design 1 (D1)				Design 2 (D2)			
	ρ = 0	ρ = .2	ρ = .5	ρ = .7	ρ = 0	ρ = .2	ρ = .5	ρ = .7
0.1	.0719	.0795	.0844	.0847	.0716	.0762	.1052	.1244
0.2	.0951	.0967	.0975	.0950	.0948	.0915	.1099	.1283
0.3	.1341	.1257	.1183	.1167	.1297	.1204	.1243	.1402
0.4	.1870	.1700	.1516	.1409	.1825	.1608	.1439	.1558
0.5	.2613	.2218	.1888	.1721	.2561	.2123	.1719	.1705
0.6	.3475	.2827	.2362	.2103	.3357	.2696	.2007	.1897
0.7	.4427	.3549	.2901	.2523	.4257	.3344	.2345	.2142
0.8	.5394	.4340	.3466	.3034	.5226	.4048	.2758	.2405
0.9	.6343	.5190	.4134	.3606	.6081	.4800	.3182	.2734
1	.7145	.5973	.4773	.4228	.6954	.5528	.3690	.3021
1.2	.8532	.7472	.6065	.5439	.8380	.6953	.4748	.3772
1.5	.9592	.8978	.7819	.7183	.9531	.8601	.6212	.4943
1.8	.9912	.9674	.9007	.8522	.9902	.9440	.7561	.6168
2	.9976	.9868	.9476	.9152	.9969	.9728	.8263	.6904
2.5	.9997	.9986	.9903	.9833	.9997	.9951	.9360	.8417
3	.9999	.9999	.9990	.9977	.9998	.9976	.9781	.9287
4	1.0000	1.0000	1	1	.9999	.9991	.9939	.9896
5	1	1	1	1	.9999	.9996	.9985	.9993
6	1	1	1	1	1	1	1	1

**Table A2:** Statistical power for Wald tests under contaminated normally distributed residuals for two intervention designs

β	Design 1 (D1)				Design 2 (D2)			
	ρ = 0	ρ = .2	ρ = .5	ρ = .7	ρ = 0	ρ = .2	ρ = .5	ρ = .7
0.1	.0560	.0657	.0865	.0954	.0553	.0758	.1142	.1258
0.2	.0651	.0698	.0903	.0932	.0611	.0774	.1092	.1265
0.3	.0789	.0864	.0966	.0995	.0772	.0858	.1140	.1297
0.4	.1016	.1009	.1014	.1015	.1018	.1042	.1207	.1314
0.5	.1264	.1163	.1170	.1158	.1250	.1156	.1272	.1374
0.6	.1521	.1432	.1260	.1188	.1517	.1463	.1460	.1431
0.7	.1870	.1667	.1473	.1324	.1885	.1678	.1472	.1510
0.8	.2334	.1922	.1633	.1465	.2280	.1942	.1685	.1604
0.9	.2712	.2294	.1853	.1687	.2668	.2142	.1837	.1673
1	.3107	.2728	.2168	.1894	.3008	.2456	.1968	.1801
1.2	.3985	.3401	.2648	.2305	.3952	.3099	.2339	.2029
1.5	.5289	.4434	.3546	.3159	.5204	.4220	.3019	.2466
1.8	.6364	.5577	.4428	.3989	.6259	.5231	.3754	.2859
2	.7074	.6142	.5025	.4494	.6911	.5720	.4131	.3317
2.5	.8259	.7427	.6462	.5889	.8213	.7100	.5287	.4190
3	.9073	.8457	.7510	.7158	.9068	.8120	.6342	.5148
4	.9775	.9482	.8910	.8657	.9730	.9343	.8041	.6952
5	.9952	.9878	.9554	.9441	.9943	.9723	.8994	.8191
6	.9991	.9962	.9857	.9781	.9981	.9875	.9459	.8987
7	.9997	.9991	.9958	.9918	.9988	.9934	.9672	.9418
8	.9999	.9999	.9987	.9973	.9990	.9948	.9789	.9628
9	1	.9997	.9999	.9990	.9991	.9960	.9833	.9762
10	1	.9998	.9999	.9995	.9998	.9973	.9894	.9813

**Table A3:** Statistical power for parametric bootstrap tests under normally distributed residuals for two intervention designs

β	Design 1 (D1)				Design 2 (D2)			
	ρ = 0	ρ = .2	ρ = .5	ρ = .7	ρ = 0	ρ = .2	ρ = .5	ρ = .7
0.1	.0591	.0552	.0553	.0574	.0556	.0527	.0518	.0558
0.2	.0753	.0640	.0621	.0635	.0736	.0606	.0559	.0573
0.3	.1118	.0853	.0754	.0723	.1091	.0821	.0672	.0605
0.4	.1524	.1233	.1011	.0925	.1519	.1100	.0790	.0628
0.5	.2187	.1663	.1247	.1174	.2186	.1432	.0899	.0722
0.6	.2936	.2168	.1597	.1409	.2860	.1856	.1098	.0891
0.7	.3781	.2804	.2049	.1836	.3745	.2364	.1296	.0962
0.8	.4638	.3425	.2471	.2172	.4586	.2874	.1501	.1181
0.9	.5658	.4215	.3042	.2705	.5474	.3410	.1780	.1368
1	.6476	.4957	.3548	.3097	.6365	.4136	.2187	.1474
1.2	.7987	.6402	.4718	.4241	.7943	.5468	.2844	.1944
1.5	.9349	.8310	.6627	.6006	.9236	.7166	.4022	.2732
1.8	.9839	.9352	.8143	.7603	.9789	.8395	.5403	.3717
2	.9935	.9660	.8882	.8453	.9930	.8935	.6146	.4446
2.5	.9997	.9969	.9727	.9585	.9994	.9672	.7948	.6115
3	.9999	.9999	.9976	.9943	.9996	.9889	.8964	.7610
4	1.0000	1.0000	1.0000	1.0000	1.0000	.9977	.9769	.9373
5	1.0000	1.0000	1.0000	1.0000	.9999	.9987	.9961	.9878
6	1.0000	1.0000	1.0000	1.0000	1.0000	.9998	.9977	.9977
7	1.0000	1.0000	1.0000	1.0000	1	.9999	.9996	.9996
8	1.0000	1	1	1	1	1.0000	.9999	.9999
9	1	1	1	1	1	1.0000	1.0000	1.0000
10	1	1	1	1	1	1	1.0000	1.0000

**Table A4:** Statistical power for parametric bootstrap tests under contaminated normally distributed residuals for two intervention designs

β	Design 1 (D1)				Design 2 (D2)			
	ρ = 0	ρ = .2	ρ = .5	ρ = .7	ρ = 0	ρ = .2	ρ = .5	ρ = .7
0.1	.0409	.0439	.0508	.0608	.0409	.0464	.0626	.0674
0.2	.0490	.0465	.0516	.0632	.0485	.0504	.0602	.0638
0.3	.0560	.0521	.0562	.0615	.0587	.0544	.0642	.0721
0.4	.0755	.0663	.0635	.0726	.0729	.0654	.0661	.0711
0.5	.0937	.0818	.0752	.0764	.0989	.0802	.0759	.0688
0.6	.1197	.0993	.0865	.0865	.1197	.0931	.0801	.0792
0.7	.1495	.1214	.0954	.0834	.1435	.1074	.0839	.0815
0.8	.1858	.1424	.1061	.1052	.1738	.1247	.0904	.0838
0.9	.2174	.1731	.1252	.1202	.2033	.1486	.1025	.0892
1	.2567	.1967	.1512	.1309	.2364	.1694	.1127	.0967
1.2	.3363	.2615	.1919	.1667	.3129	.2160	.1293	.1031
1.5	.4546	.3574	.2581	.2232	.4209	.3066	.1710	.1315
1.8	.5739	.4714	.3408	.2998	.5319	.3852	.2178	.1566
2	.6253	.5318	.4099	.3501	.6079	.4552	.2570	.1847
2.5	.7674	.6743	.5522	.4937	.7468	.5943	.3598	.2478
3	.8628	.7798	.6638	.6235	.8469	.7089	.4478	.3245
4	.9631	.9143	.8327	.8053	.9426	.8565	.6377	.4743
5	.9889	.9719	.9310	.9129	.9830	.9297	.7673	.6237
6	.9981	.9908	.9720	.9609	.9939	.9685	.8503	.7493
7	.9995	.9971	.9898	.9847	.9959	.9846	.9168	.8400
8	.9999	.9996	.9964	.9926	.9981	.9908	.9481	.8930
9	1	.9999	.9989	.9974	.9985	.9932	.9682	.9320
10	1	1	.9990	.9991	.9992	.9960	.9803	.9575

**Table A5:** Statistical power for semi‐parametric bootstrap tests under normally distributed residuals for two intervention designs

β	Design 1 (D1)				Design 2 (D2)			
	ρ = 0	ρ = .2	ρ = .5	ρ = .7	ρ = 0	ρ = .2	ρ = .5	ρ = .7
0.1	.0577	.0478	.0509	.0481	.0523	.0501	.0507	.0535
0.2	.0729	.0678	.0663	.0605	.0674	.0625	.0588	.0531
0.3	.1018	.0929	.0816	.0769	.0990	.0834	.0659	.0581
0.4	.1489	.1211	.1007	.0930	.1429	.1031	.0722	.0647
0.5	.2094	.1604	.1285	.1176	.1961	.1444	.0881	.0720
0.6	.2828	.2207	.1688	.1480	.2629	.1803	.1109	.0828
0.7	.3649	.2769	.2026	.1786	.3395	.2321	.1358	.0957
0.8	.4535	.3333	.2401	.2140	.4227	.2881	.1560	.1122
0.9	.5435	.4241	.3020	.2686	.5042	.3517	.1877	.1271
1	.6317	.4876	.3531	.3153	.5870	.4080	.2142	.1449
1.2	.7786	.6488	.4814	.4331	.7377	.5374	.2880	.1927
1.5	.9189	.8261	.6671	.6053	.8819	.7144	.4201	.2795
1.8	.9780	.9287	.8157	.7669	.9537	.8378	.5427	.3778
2	.9913	.9645	.8897	.8471	.9767	.8951	.6241	.4505
2.5	.9996	.9961	.9781	.9634	.9955	.9670	.7982	.6259
3	.9999	.9994	.9972	.9946	.9994	.9899	.9029	.7709
4	1	1	.9999	.9999	1	1	.9816	.9423
5	1	1	1	1	1	1	.9958	.9895
6	1	1	1	1	1	1	1	.9982
7	1	1	1	1	1	1	1	.9998
8	1	1	1	1	1	1	1	1
9	1	1	1	1	1	1	1	1
10	1	1	1	1	1	1	1	1

**Table A6:** Statistical power for semi‐parametric bootstrap tests under contaminated normally distributed residuals for two intervention designs

β	Design 1 (D1)				Design 2 (D2)			
	ρ = 0	ρ = .2	ρ = .5	ρ = .7	ρ = 0	ρ = .2	ρ = .5	ρ = .7
0.1	.0475	.0464	.0541	.0574	.0422	.0448	.0550	.0619
0.2	.0582	.0568	.0537	.0554	.0483	.0458	.0597	.0617
0.3	.0660	.0558	.0614	.0575	.0585	.0504	.0577	.0590
0.4	.0788	.0677	.0649	.0620	.0742	.0605	.0606	.0610
0.5	.1027	.0903	.0699	.0699	.0936	.0716	.0649	.0627
0.6	.1261	.0964	.0847	.0783	.1144	.0855	.0718	.0654
0.7	.1596	.1222	.0951	.0877	.1409	.0994	.0774	.0684
0.8	.1842	.1500	.1132	.0968	.1701	.1159	.0841	.0736
0.9	.2293	.1789	.1328	.1095	.2051	.1358	.0924	.0753
1	.2721	.2008	.1423	.1232	.2433	.1606	.1007	.0805
1.2	.3420	.2690	.1950	.1559	.3149	.2079	.1215	.0917
1.5	.4711	.3743	.2638	.2140	.4282	.2890	.1587	.1113
1.8	.5805	.4747	.3468	.2851	.5355	.3760	.1992	.1359
2	.6509	.5420	.3935	.3358	.5978	.4271	.2299	.1543
2.5	.7828	.6708	.5315	.4644	.7384	.5569	.3157	.2105
3	.8705	.7869	.6564	.5865	.8305	.6692	.4105	.2774
4	.9675	.9223	.8331	.7810	.9338	.8233	.5635	.4132
5	.9923	.9713	.9218	.8945	.9746	.9083	.7016	.5467
6	.9975	.9914	.9688	.9531	.9887	.9487	.8083	.6690
7	.9998	.9980	.9877	.9826	.9957	.9727	.8671	.7663
8	1	.9994	.9969	.9935	.9975	.9822	.9187	.8343
9	1	.9999	.9981	.9973	.9981	.9889	.9446	.8848
10	1	1	.9997	.9989	.9985	.9924	.9626	.9234
